# Sevoflurane Induces DNA Damage Whereas Isoflurane Leads to Higher Antioxidative Status in Anesthetized Rats

**DOI:** 10.1155/2015/264971

**Published:** 2015-05-25

**Authors:** Thalita L. A. Rocha, Carlos A. Dias-Junior, Jose S. Possomato-Vieira, Victor H. Gonçalves-Rizzi, Flávia R. Nogueira, Kátina M. de Souza, Leandro G. Braz, Mariana G. Braz

**Affiliations:** ^1^Department of Pharmacology, Biosciences Institute of Botucatu, Sao Paulo State University (UNESP), Distrito de Rubião Júnior, s/n, 18618-000 Botucatu, SP, Brazil; ^2^Department of Anesthesiology, Botucatu Medical School, Sao Paulo State University (UNESP), Distrito de Rubião Júnior, s/n, 18618-970 Botucatu, SP, Brazil

## Abstract

Taking into account that there are controversial antioxidative effects of inhalational anesthetics isoflurane and sevoflurane and absence of comparison of genotoxicity of both anesthetics in animal model, the aim of this study was to compare DNA damage and antioxidant status in Wistar rats exposed to a single time to isoflurane or sevoflurane. The alkaline single-cell gel electrophoresis assay (comet assay) was performed in order to evaluate DNA damage in whole blood cells of control animals (unexposed; *n* = 6) and those exposed to 2% isoflurane (*n* = 6) or 4% sevoflurane (*n* = 6) for 120 min. Plasma antioxidant status was determined by 3-(4,5-dimethylthiazol-2-yl)-2,5-diphenyltetrazolium bromide (MTT) assay. There was no statistically significant difference between isoflurane and sevoflurane groups regarding hemodynamic and temperature variables (*P* > 0.05). Sevoflurane significantly increased DNA damage compared to unexposed animals (*P* = 0.02). In addition, Wistar rats anesthetized with isoflurane showed higher antioxidative status (MTT) than control group (*P* = 0.019). There were no significant differences in DNA damage or antioxidant status between isoflurane and sevoflurane groups (*P* > 0.05). In conclusion, our findings suggest that, in contrast to sevoflurane exposure, isoflurane increases systemic antioxidative status, protecting cells from DNA damage in rats.

## 1. Introduction

The possibility of health hazards resulting from exposure to volatile anesthetics has been extensively discussed during the last decade. There are some epidemiological data suggesting neurotoxic, hepatotoxic, and nephrotoxic side effects from inhalational anesthetics [1, 2]. Several studies have pointed out genetic damage in operating room personnel exposed to trace concentrations of anesthetic gases [3–5].

Isoflurane (C_3_H_2_ClF_5_O) and sevoflurane (C_4_H_3_F_7_O) are inhalational anesthetics widely used in current clinical practice. Both halogenated anesthetics have advantages because of low blood-gas partition coefficients, being sevoflurane with lower solubility (0.65) than isoflurane (1.4), allowing rapid induction and awakening from anesthesia [6].

The genotoxicity and mutagenicity of isoflurane have been evaluated* in vitro* and in clinical studies showing conflicting results [7–9]. Similar findings are described for sevoflurane [10–12]. Literature is scarce regarding the possible genotoxic effects of isoflurane or sevoflurane in experimental studies. Moreover, no report yet has compared the genotoxicity of isoflurane and sevoflurane in animal model.

So far, the alkaline single-cell gel electrophoresis assay, also known as comet assay, has been extensively used to determine the extent of DNA damage, including strand breaks, alkali-labile sites, DNA cross-linking, and incomplete excision repair sites in mammalian cells [13]. Fragments of DNA migrate farther in response to an electric field, so that the nucleoids resemble a “comet” with a brightly fluorescent head and tail region [14]. This is a rapid, simple, sensitive, and reliable biochemical technique for evaluating DNA injury after exposure to toxicants.

It is still controversial whether the volatile anesthetics lead to oxidative stress. Many reports on occupational exposure to anesthetics have shown they can impair antioxidant status [5, 15, 16]. On the other hand, some clinical studies have shown volatile anesthetics do not alter redox status [12, 17].

Because of the absence of reports on genotoxicity together with controversial antioxidative effects of halogenated anesthetics* in vivo*, the aim of the current study was to compare systemic DNA damage and antioxidant status in rats exposed to either isoflurane or sevoflurane, without undergoing surgery procedure.

## 2. Materials and Methods

### 2.1. Animals

This study was approved by the Ethical Committee for Animal Research (protocol number 684) from the Biosciences Institute of Botucatu from Sao Paulo State University (UNESP). All animals were treated in accordance with the recommendations of the Ethical Principles approved by the Brazilian Society of Science in Laboratory Animals.

A total of 18 male Wistar rats, weighing 300–350 g, were provided by the Biosciences Institute of Botucatu, UNESP. The animals were maintained at the Department of Pharmacology (UNESP) with restricted-access rooms at a controlled temperature (23 ± 2°C) and on a 12 h light-dark cycle. The animals were given free access to a standard chow diet and drinking water* ad libitum*, and their age was 10 weeks on the day of exposure.

### 2.2. Experimental Design

The rats were assigned randomly to one of three groups, each of which consisted of 6 animals that were unexposed (control, C group) or exposed to different volatile anesthetics: isoflurane (*Isoforine*, Cristalia, Sao Paulo, Brazil; ISO group) or sevoflurane (*Sevorane*, Abbott, Buenos Aires, Argentina; SEVO group).

Isoflurane and sevoflurane concentrations were recorded by means of an infrared analyzer (Vamos Plus; Dräger, Lübeck, Germany). Induction of anesthesia with 3% isoflurane or 5% sevoflurane at a continuous oxygen flow (2 L/min) was performed in a glass chamber connected to an anesthesia machine (AI; Insight, Ribeirao Preto, Brazil). Having confirmed immobility and loss of righting reflex, the animals were placed in ventral recumbency on heat pad for preventing hypothermia (Heat Pad; Insight, Ribeirao Preto, Brazil). The anesthetic plan was maintained by face mask with 2% isoflurane or 4% sevoflurane.

**Figure 1:**
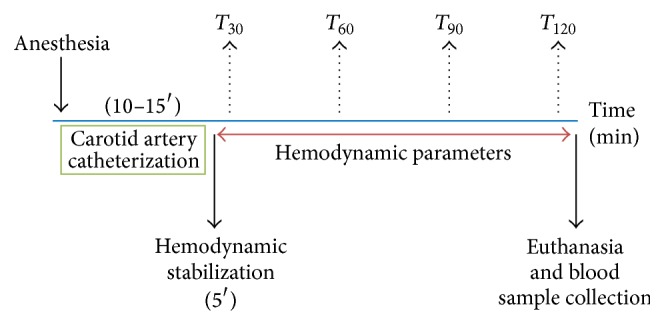
Experimental design for rats exposed to 2% isoflurane or 4% sevoflurane for 120 min.

Subsequently, a polyethylene catheter (PE50) was inserted into the left carotid for evaluation of the mean, systolic, and diastolic arterial pressure. Data were recorded using a data acquisition system (MP150CE; Biopac Systems Inc., Goleta, CA) connected to a computer (Acknowledge 3.2, for Windows). Heart rate values were derived from the blood pressure recordings and processed online. The absence of somatic motor reflexes in response to tail pitching or blinking in response to a low-pressure corneal stimulation indicated deep anesthesia and analgesia. Body temperature was measured using a probe inserted in the rectum of each rat, which was connected to a monitor (DX 2023 monitor; Dixtal Biomedica, Sao Paulo, Brazil). The data acquisitions were initiated 30 min after anesthesia onset [18]. The experimental design for anesthetized rats is presented in Figure 1.

### 2.3. Blood Collection

Blood was collected in EDTA tubes from each decapitated rat from all groups, and comet assay was carried out immediately. Part of the blood was centrifuged to obtain the plasma, which was aliquoted and stored until evaluation of antioxidant status (MTT assay). All the procedures were performed under dim light to prevent additional DNA damage.

### 2.4. Genotoxicity Assay

Before performing comet assay, cell viability was determined by trypan blue dye exclusion [19]. The protocol used for the alkaline single-cell gel electrophoresis assay (comet assay) followed the guidelines previously proposed [13]. Briefly, 10 *μ*L of fresh peripheral blood cells was added to 100 *μ*L of 0.5% low-melting point agarose at 37°C, layered onto a precoated slide with 1.5% regular agarose in duplicate, and covered with a coverslip. After brief agarose solidification in a refrigerator, the coverslip was removed and slides were immersed in lysis solution (2.5 M NaCl, 100 mM EDTA, and 10 mM Tris, pH 10, with 1% Triton X-100 and 10% dimethyl sulfoxide) overnight. Slides were then washed in phosphate-buffered saline (PBS) for 5 min and immersed in a freshly prepared alkaline buffer (pH 1 mM EDTA and 300 mM NaOH, pH > 13) for 20 min and the electrophoresis was carried out using the same solution conducted for 20 min at 25 V and 300 mA. Following this step, the slides were neutralized in 0.4 M Tris-HCl (pH 7.5) for 15 min, fixed in absolute ethanol for 5 min, and stored at room temperature until analysis. The slides were stained with Sybr Gold and a total of 100 randomly captured nucleoids per animal (50 from each slide) were examined blindly by one expert observer at 400x magnification using a fluorescent microscope connected to an image analysis system (Comet Assay IV, Perceptive Instruments, UK) that was calibrated previously according to the manufacturer's instructions. The parameter tail moment was considered to measure DNA damage (arbitrary units).

### 2.5. Evaluation of Antioxidant Status

Direct reductions in MTT (3-(4,5-dimethylthiazol-2-yl)-2,5-diphenyltetrazolium bromide) were measured as previously described [20] with slight modifications. Briefly, 100 *μ*L of plasma was mixed with 12.5 *μ*L of dye solution (5 mg/mL in PBS); the final volume was adjusted to 200 *μ*L with PBS, and the mixture was incubated for 60 min at 37°C. The reaction was terminated by the addition of 750 *μ*L of 0.04 M hydrochloric acid in isopropanol. The tubes were centrifuged for 10 min at 1000 ×g, the supernatant was collected, and the absorbance was measured at 570 nm.

### 2.6. Statistical Analysis

Hemodynamic and temperature data were compared between isoflurane and sevoflurane groups and within each group using ANOVA followed by a Tukey test or the *t*-test. For body weight, comet assay, and MTT data, since they showed a normal distribution, ANOVA was applied to compare the three groups, followed by Tukey test, when necessary. A probability value *P* < 0.05 was considered statistically significant.

## 3. Results

Table 1 shows no statistically significant difference between isoflurane and sevoflurane groups regarding hemodynamic variables (*P* > 0.05). In addition, body weight did not statistically differ among groups, and rectal temperature data did not differ between isoflurane and sevoflurane groups (data not shown; *P* > 0.05). None of the animals died during anesthesia.

**Figure 2:**
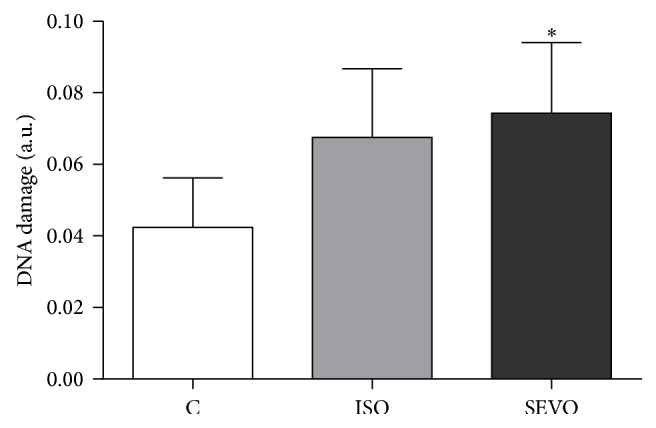
DNA damage (mean ± S.D.) detected by comet assay in whole blood cells of animals unexposed to anesthetics (C = control) or exposed to 2% isoflurane (ISO) or 4% sevoflurane (SEVO).  ^*∗*^ *P* = 0.02* versus* control.

Cell viability was higher than 98% for all groups (99.7% for control group, 98.2% for isoflurane group, and 98.9% for sevoflurane group). The results of the comet assay in the peripheral blood of rats are shown in Figure 2. Sevoflurane significantly increased DNA damage compared to the control (*P* = 0.02). DNA damage was slightly higher in isoflurane group compared to the control, but with no significant difference (*P* > 0.05). No significant differences regarding DNA damage were found between isoflurane and sevoflurane groups (*P* > 0.05).

**Figure 3:**
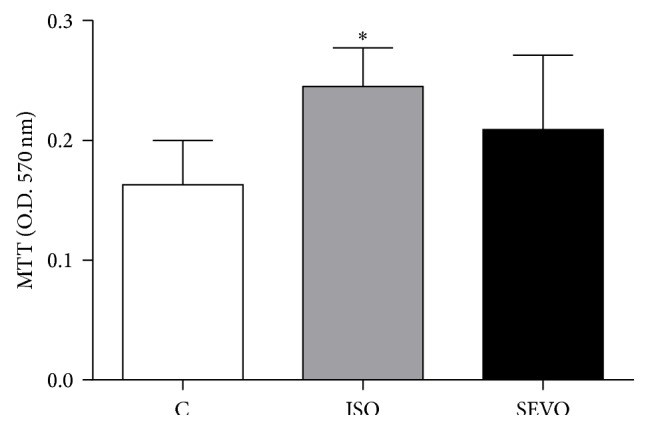
Plasma antioxidant status (mean ± S.D.) of animals unexposed to anesthetics (C = control) or exposed to isoflurane (ISO) or sevoflurane (SEVO).  ^*∗*^ *P* = 0.019* versus* control.

Rats anesthetized with isoflurane showed higher antioxidative status (MTT) than control group (*P* = 0.019). There was no significant difference regarding antioxidant capacity between isoflurane and sevoflurane groups (*P* > 0.05; Figure 3).

## 4. Discussion

The main findings of the current study are that sevoflurane induced DNA damage whereas isoflurane led to a higher antioxidative status in Wistar rats exposed for 120 min.

The maintenance of hemodynamic stability and rectal temperature were similar in both anesthetics and were relevant since alterations in these parameters may influence the results.

The novelty of this study consists of comparing two different halogenated anesthetics widely used, in rats exposed to a single time for 120 min. Thus, different from clinical practice, we can isolate the role of anesthetic agents from surgery to try to understand the systemic effects of these drugs. Information about genotoxicity of modern halogenated anesthetics is still insufficient. Thus, the current study indicates for the first time that rats exposed once to sevoflurane have increased systemic genetic damage within a few hours, when compared to unexposed animals. The concentrations of 2% and 4% of isoflurane and sevoflurane, respectively, have already been used in Wistar rats, allowing an adequate anesthesia plan [21–23].

Male mice repeatedly exposed to 2.4% sevoflurane (2 h daily, for 3 days) presented more DNA damage in leukocytes detected by comet assay and blood micronucleus compared to the control [24]. Repeated sevoflurane anesthesia (3% in oxygen for 3 h/day for 3 consecutive days) was investigated in male rabbits with or without antioxidant supplementation [25]. The authors found that previously vitamin E (50 I.U./day) or selenium (15 *μ*g/day) supplementation prevented increase of DNA damage in mononuclear cells when compared to nonsupplemented animals exposed to sevoflurane.

Some advantages of sevoflurane in clinical practice include the very low blood and tissue solubility and a pleasant odor. However, about 5% of inhaled sevoflurane is metabolized in the liver by cytochrome P450 2E1 isoenzyme, giving rise to reactive products, which could directly trigger the generation of peroxynitrite and increase peroxides and nitric oxide [26]. It is known that free radicals or reactive oxygen species (ROS) are major oxidants that react with DNA, damaging it by various lesions, such as oxidized bases, abasic sites, and/or strand breaks [27]. Some authors have also suggested that fluorinated anesthetics, including sevoflurane, could directly lead to DNA damage, and the most probable modification would be an alkylation of purines [8]. Additionally, sevoflurane can induce cellular apoptosis [28]. The observed increase of DNA damage in sevoflurane group may be due to genotoxicity, and not to cell toxicity, since the exposure did not decrease cell viability. Thus, possible mechanisms of sevoflurane genotoxicity include direct genotoxicity and/or oxidative route by metabolism.

Induced DNA damage occurred in blood cells earlier than tissues such as liver, kidney, and brain [24]. According to the authors, blood is the first compartment to absorb sevoflurane and the hematopoietic system may be highly sensitive to genotoxic agents, in part because hematopoietic cells undergo rapid division.

However, a few negative results concerning sevoflurane genotoxicity have already been reported. This anesthetic was not able to induce genetic lesions* in vitro*, when lymphocytes were exposed to 1 mM or 10 mM at 4°C or 37°C for 10 and 30 min [29]. No changes in oxidative DNA damage were observed in adults without comorbidities who underwent minimally invasive surgeries maintained with 1.9% sevoflurane anesthesia [12].

Different from isoflurane, sevoflurane did not enhance plasma antioxidative status in exposed rats. Similar results were described in literature. A study showed that sevoflurane had no effects on the antioxidant system (glutathione peroxidase and superoxide dismutase enzymes) of anesthetized pigs [30]. Other study revealed that sevoflurane anesthesia did not alter the activities of antioxidant enzymes in the liver, brain, and lung of exposed rats [31]. Additionally, no changes in glutathione peroxidase and catalase activities in rat erythrocytes were detected after 4% sevoflurane exposure [22].

The International Agency for Research on Cancer (IARC) stated that there is inadequate evidence for the carcinogenicity of isoflurane in animals. Volatile anesthetics are not classifiable as to their carcinogenicity to humans [32]. In the current study, we did not observe a significant difference between comet assay data in isoflurane and sevoflurane groups. Interestingly, any difference was also observed in systemic DNA damage when adult patients were anesthetized with isoflurane or sevoflurane [33]. Supporting our findings, isoflurane was not found mutagenic when evaluated in the bacterial Ames test, using metabolic activation or not, or in* Drosophila melanogaster* [34, 35].

Contrarily, repeated exposure to isoflurane (1.7% in oxygen for 2 h daily for 3 consecutive days) induced genotoxicity in leukocytes and some organs of 8-week-old male Swiss albino mice [21]. Sprague-Dawley rats exposed to isoflurane (1% in air for 30 min or 60 min) have increased time-dependent DNA damage detected in lymphocytes [36]. In contrast, our study showed isoflurane did not increase DNA damage. Differences in isoflurane anesthesia have already been reported in Wistar and Sprague-Dawley rats [37]. Thus, besides animal strain, time (30 or 60 min* versus* 120 min) and concentration of exposure (1%* versus* 2%), animal age (6–8 weeks* versus* 10 weeks), and target cells analyzed (isolated lymphocytes* versus* whole blood) are some factors that could explain opposite findings concerning genotoxicity between the studies. Regarding comet assay, we evaluated DNA damage in whole blood cells. Among the advantages of using peripheral blood are the speed, the low cost, and the simplicity in performing the assay and the lower variability of the results [38–40].

It also must be highlighted that, different from sevoflurane, hepatic biotransformation of isoflurane is low (≤0.2%) [41]. Clinical studies performed by our research group indicated absence of systemic DNA breaks or oxidative DNA damage in patients under isoflurane anesthesia [9, 17].

Despite the increase of oxidative stress parameters such as lipid and protein oxidation in rats exposed to isoflurane for 60 min, Kim et al. [36] detected any alteration during the first 30 min. The authors could not show evidence of an association between DNA damage and oxidative stress parameters. Differently, in the current study, we have shown rats anesthetized with isoflurane presented higher plasma antioxidative status. Interestingly, patients undergoing minimally invasive surgery lasting 120 min showed slight increase of plasma antioxidant capacity during isoflurane anesthesia [17]. Much is still unknown about the possible mechanisms by which isoflurane can have antioxidative properties. It has already been reported that anesthetics can modulate heme oxygenase- (HO-) 1, which exerts anti-inflammatory and antioxidative effects [42]. Isoflurane can induce HO-1 via nuclear factor kappa B (NF*κ*B) [43].

It is already known that anesthetic preconditioning and protection from tissue ischemic injury involve ROS, but the mechanisms are unknown [44]. Thus, isoflurane may provide a benefit against ischemia-reperfusion (IR) injury. A study provided evidence that induction of the cytoprotective enzyme HO-1 by nontoxic and clinically approved isoflurane concentration protected rat livers from IR injury [45]. This anesthetic can attenuate oxidative stress and has neuroprotective effects* in vitro*, but it may work through indirect mechanisms to reduce oxidative stress-induced cell injury [46]. The pretreatment with isoflurane protected cardiomyocytes from damage by oxidative stress; sarcolemmal and mitochondrial Adenosine Triphosphate- (ATP-) sensitive potassium channels play essential and distinct roles in protection afforded by this anesthetic [47]. In addition, isoflurane reduced myocardial infarction size by modulating mitochondrial ROS at clinical concentrations [44]. Certainly further investigations are required to better comprehend the possible mechanisms of antioxidant capacity of isoflurane, especially in a non-IR injury model.

## 5. Conclusions

Under the established conditions, this investigation provides evidence that, in contrast to sevoflurane exposure, isoflurane increases systemic antioxidative status, which can protect cells from DNA damage in rats.

## Figures and Tables

**Table 1:** Hemodynamic variables in anesthetized rats with 2% isoflurane or 4% sevoflurane (ISO and SEVO, resp.). Data were recorded at 30 (*T*_30_), 60 (*T*_60_), 90 (*T*_90_), and 120 min (*T*_120_) of anesthesia. Data are presented as mean (S.D.). HR: heart rate; SAP: systolic arterial pressure; MAP: mean arterial pressure; DAP: diastolic arterial pressure. *P* > 0.05 among time points in the same group and between groups regarding a specific time point.

Variables	Groups	Time points			
		*T*_30_	*T*_60_	*T*_90_	*T*_120_
Heart rate (beats/min)	ISO	312 (30)	326 (30)	327 (44)	324 (46)
	SEVO	323 (45)	338 (32)	357 (26)	365 (29)

SAP (mm Hg)	ISO	87 (13)	90 (9)	87 (14)	86 (16)
	SEVO	103 (19)	98 (15)	98 (12)	105 (18)

MAP (mm Hg)	ISO	78 (9)	84 (6)	80 (9)	79 (12)
	SEVO	89 (21)	84 (17)	84 (12)	87 (17)

DAP (mm Hg)	ISO	67 (10)	73 (7)	73 (5)	65 (11)
	SEVO	76 (20)	70 (15)	71 (11)	74 (15)
